# Prevalence, awareness, treatment, control and socio demographic determinants of hypertension in Malaysian adults

**DOI:** 10.1186/s12889-016-3008-y

**Published:** 2016-04-21

**Authors:** Suraya Abdul-Razak, Aqil Mohammad Daher, Anis Safura Ramli, Farnaza Ariffin, Md Yasin Mazapuspavina, Krishnapillai S. Ambigga, Maizatullifah Miskan, Hasidah Abdul-Hamid, Nafiza Mat-Nasir, Mohamed Noor Khan Nor-Ashikin, Kien Keat Ng, Hapizah Nawawi, Khalid Yusoff, for the REDISCOVER Investigators

**Affiliations:** Faculty of Medicine, Universiti Teknologi MARA, Selayang Campus, Jalan Prima Selayang 7, 68100 Batu Caves, Selangor Malaysia; Institute of Pathology, Laboratory and Forensic Medicine (I-PPerForM), Universiti Teknologi MARA, Selayang Campus, Jalan Prima Selayang 7, 68100 Batu Caves, Selangor Malaysia; Faculty of Medicine & Defence Health, National Defence University of Malaysia, 57000 Kuala Lumpur, Malaysia; Centre for Translational Research and Epidemiology (CenTRE), Faculty of Medicine, Universiti Teknologi MARA, 47000 Sungai Buloh, Selangor Malaysia; UCSI University, UCSI Height, Cheras, 56000 Kuala Lumpur, Malaysia; Primary Care Medicine Discipline, Universiti Teknologi MARA, 68100 Selayang, Selangor Malaysia

**Keywords:** Prevalence, Awareness, Treatment, Control, Hypertension, Malaysia

## Abstract

**Background:**

Hypertension is the leading cardiovascular risk factor globally as well as in Malaysia. This study aimed to estimate the prevalence, awareness, treatment, control and the socio demographic determinants of hypertension among Malaysian adults.

**Method:**

The analytic sample consisted of 11,288 adults aged ≥ 30 years recruited at baseline in 2007–2011 from the REDISCOVER Study which is an ongoing, prospective cohort study involving 18 urban and 22 rural communities in Malaysia. Socio-demographics, anti-hypertensive treatment details and an average of at least two blood pressure measurements were obtained.

**Results:**

The age-adjusted prevalence was 42.0 % (CI: 40.9–43.2) and was higher in men [43.5 % (CI: 41.2–45.0)] than women [41.0 % (CI: 39.8–42.3)]. Participants from rural areas (APR: 1.12, CI: 1.04–1.20); aged at least 40–49 years (APR: 1.86, CI: 1.62–2.14); who were overweight (APR: 1.24, CI: 1.15–1.34) and obese (APR: 1.54, CI: 1.43–1.6) were more likely to have hypertension. The Indigenous ethnic group was less likely to be aware (APR: 0.81, CI: 0.69–0.92) and to be on treatment (APR: 0.66, CI: 0.55–0.79). Those in rural areas were less likely to have their hypertension controlled (APR: 0.61, CI: 0.49–0.75). On the other hand, control was more likely in females (APR: 1.25, CI: 1.01–1.54) and Indigenous group (APR: 1.64, CI: 1.19–2.25).

**Conclusion:**

Hypertension is common in the Malaysian adults. The control of hypertension has increased over the years but is still quite low. Public health measures, as well as individual interventions in primary care are crucial to reduce their risk of developing complications.

## Background

Hypertension is the number one cardiovascular risk factor and the leading cause of mortality worldwide [1]. Malaysia, like other developing countries is experiencing an upsurge in cardiovascular morbidity and mortality [2]. The emergence of cardiovascular disease as a leading cause of death in Malaysia runs parallel with the rapid economic growth and associated socio-demographic change that has occurred over the past few decades. Thus, achieving blood pressure (BP) control and prevention of cardiovascular morbidity and mortality is vital and should be strived for, as many effective and inexpensive BP treatments options are now available.

Data on prevalence, awareness, treatment and control in various communities are necessary for monitoring and developing new strategies for hypertension control. A few reports have documented the national prevalence, awareness and treatment rates in Malaysia [2–5]. A national health morbidity survey (NHMS) conducted in 2011 among adults aged ≥18 years reported that the overall prevalence of hypertension was 32.7 % and the treatment rate of those who were aware was 78.4 % [4]. Another study conducted a decade ago reported that the overall prevalence, treatment and control among individuals aged ≥15 years were 27.8 %, 32.4 % and 8.6 % respectively [3]. Although local data on the prevalence, awareness, treatment and control of hypertension were available, data on variation between urban and rural settings and body mass index (BMI) are still lacking. Such information is vital to guide the allocation of resources towards developing strategies for better detection and control of hypertension in Malaysia. Therefore, this study aimed to evaluate the prevalence, treatment, awareness and control of hypertension in Malaysia and the association with socio-demographic factors including urban–rural and BMI variations.

## Methods

Sampling methods: The REDISCOVER Study is an ongoing prospective cohort study involving Malaysian adults aged ≥ 30 years from 18 urban and 22 rural communities from the states of Selangor, Negeri Sembilan, Pahang, Kelantan, Sarawak and Sabah, and the Federal Territory of Kuala Lumpur. Participants were selected in a four-stage sampling process: selecting the states and then the ‘communities’, followed by households within them and finally individuals within the households. The 5 states were chosen to ensure adequate representation of the major ethnic groups in Malaysia. A standardized method of recruitment was adopted. All household members aged ≥ 30 years were invited to attend screening sessions in local community centres. Approximately, 20 000 invitations were sent out. A response rate of 60–70 % was recorded at each site. At the screening sites, participants were given information leaflet about the study and were screened for eligibility. Written informed consent was obtained from those who were eligible and willing to participate.

The baseline data was collected from 2007 to 2011. The study duration is 15 years and data collection is repeated every three years. The cross-sectional analytic sample presented in this paper consisted of 11,288 participants who were recruited at baseline. A detailed description of the design and methodology of this study has been published elsewhere [6]. The institutional ethics committee approved the study protocol.

Study procedures: All interviewers and investigators were trained regarding the study procedures prior to the conduct of the study in order to standardize the data collection and to minimize variability during data collection. Standardized, pre-tested, interviewer-based questionnaires were used to collect information regarding age, gender, ethnic group, educational level, smoking status, and known history and treatment of hypertension.

The blood pressure was measured at least twice at five minutes apart on the right arm supported at heart level, using Omron automatic digital blood pressure monitor (Omron HEM-757). Participants were advised not to smoke, exercise or eat in the last 30 min, not to climb stairs in the last 15–30 min, and were made to rest for at least five minutes before the measurements were taken. The average of the two BP readings was used for analysis. If the measurements differ by 5 mmHg of either systolic or diastolic readings, subsequent measurements were taken at 5–10 min apart. The process was repeated until two BP values, which did not differ by more than 5 mmHg of either systolic or diastolic readings, were obtained. The average of these two BP readings was used as the BP value for that particular subject.

Definitions of socio-demographic factors: Urban and rural areas were defined according to the Malaysian Population and Housing Census 2000 [7]. Gazetted areas with a combined population of 10,000 or more were identified as urban areas and all the other areas with a population of less than 10,000 were classified as rural. Ethnic groups were categorized as Malays, Chinese, Indians and Indigenous. Kadazan-Dusun, Bajau, Murut and several other ethnic minorities who live in East Malaysia represented the Indigenous group. Education attainment levels were classified into four categories as ‘no formal education’, ‘primary’, ‘secondary’ and ‘tertiary’. Participants who had never been to school to get any form of education were categorised into ‘no formal education’, while ‘primary’ education level represented those with at least 7 years of schooling at primary school. ‘Secondary’ education level represented those with at least 5 years of schooling at secondary school, whereas ‘tertiary’ education level represented those who attended colleges or universities. Current smokers were defined as participants who were currently smoking cigarettes or had smoked cigarettes within the past five years. Ex- smokers were those who had stopped smoking for more than five years and non-smokers were those who had never smoked. BMI was classified according to the Malaysia Guideline on the Management of Obesity, 2004 [8]. Underweight was defined as BMI < 18.5 kg/m^2^, normal range as BMI of 18.5–22.9 kg/m^2^, overweight as BMI of 23–27.4 kg/m^2^ and obesity as BMI ≥ 27.5 kg/m^2^.

Definition of hypertension, awareness, treatment and control: Hypertension was defined according to the Malaysia Guideline on the Management of Hypertension, 3^rd^ Edition, 2008. “Hypertension” was considered to be present if: (1) the average systolic BP ≥ 140 mmHg and/or average diastolic BP ≥ 90 mmHg; (2) or the participants reported a history of hypertension; (3) or participants reported taking anti-hypertensive medications in the past two weeks. “Awareness of hypertension” was defined as self-report of any previous diagnosis of hypertension by a healthcare professional among those with hypertension. “Treatment of hypertension” was defined as self-reported use of antihypertensive medications among those with hypertension. “Control among hypertensive participants” was defined as having a BP < 140/90 mmHg among those with hypertension.

### Data analysis

Our study population was described in terms of its socio-demographic characteristics using simple descriptive statistics. Data were presented as percentages for categorical variables and numerical variables were described with mean (± Standard Deviation [SD]). The overall prevalence of hypertension, awareness, treatment and control were described for the total study population with 95 % confidence interval (CI). The prevalence of hypertension was also age standardized using World Health Organisation (WHO) world population for people aged 30 years and above. The modified Poisson regression model with robust variance was used to estimate the crude and adjusted prevalence ratio [9, 10] in this study. To identify independent factors associated of being hypertensive, aware, treated and controlled; location, gender, age, ethnicity, education attainment, smoking status and BMI were controlled for each other using the modified Poisson regression model. The crude prevalence ratios (CPR) were compared to adjusted prevalence ratios (APR) and their 95 % CI. A two-sided P-value of <0.05 was considered to be statistically significant. All analyses were performed using STATA software version 11.1 with (StataCorp.TX).

## Results

### Characteristics of the respondents

A total of 11 288 eligible adults participated in this study. Table 1 shows the socio-demographic characteristics of the participants by locality, age, gender, ethnicity, education attainment, smoking status and BMI. The mean age was 53.02 (SD ±10.9) years. There were more participants from urban areas (51.9 %) and females (56.2 %). Majority of the participants were Malays (72.5 %), followed by Indigenous group (13.8 %), Chinese (10.8 %) and Indians (2.9 %). In terms of education attainment, 38.4 % of the participants had secondary education level and majority had never smoked cigarette (75.6 %). The proportions of participants who were overweight and obese were 38.7 % and 34.3 %, respectively.

**Table 1:** Demographic characteristics

Demographic characteristics	
All subjects (*n*, %)	11,288 (100)
Mean age in years (±SD)	53.02 (±10.9)
Gender (*n*, %)	
Male	4943 (43.8)
Female	6345 (56.2)
Location (*n* ^a^, %)	
Urban	5857 (51.9)
Rural	5410 (48.1)
Age (years) (*n*, %)	
30–39	1233 (10.9)
40–49	3336 (29.6)
50–59	3606 (31.9)
≥60	3113 (27.6)
Ethnicity (*n*, %)	
Malay	8188 (72.5)
Chinese	1214 (10.8)
Indian	327 (2.9)
Indigenous group	1559 (13.8)
Education attainment (*n* ^a^, %)	
No formal education	1566 (15.3)
Primary	2766 (27.0)
Secondary	3929 (38.4)
Tertiary	1980 (19.3)
Smoking status (*n* ^a^, %)	
Non smoker	8014 (75.6)
Current smoker	1367 (12.9)
Ex-smoker	1224 (11.5)
Body Mass Index (*n* ^a^, %)	
Underweight	392 (3.7)
Normal	2493 (23.3)
Overweight	4144 (38.7)
Obese	3670 (34.3)

*n* ^*a*^ is not equal to 11 288 due to missing values

### Prevalence of hypertension, awareness, treatment and control

Table 2 shows the overall prevalence of hypertension, awareness, treatment, and control by locality, gender, ethnicity, education attainment, smoking status and BMI. The overall prevalence of hypertension in our sample was 47.9 % (CI: 47.0–49.0). The age-adjusted prevalence was 42.0 % (CI: 40.9–43.2) and was higher in men [43.5 % (CI: 41.2–45.0)] than women [41.0 % (CI: 39.8–42.3)]. Out of those who have hypertension, 53.2 % (CI: 51.9–54.5) were aware, 38.2 % (CI: 36.9–39.5) were on treatment, and 15.9 % (CI: 14.9–16.9) had their BP controlled. Out of those who were aware, 72.3 % (CI: 70.6–73.9) received treatment and 30.3 % (CI: 28.7–32.0) had their BP controlled. Out of those who were treated, 30.7 % (CI: 28.7–32.7) achieved BP control.

**Table 2:** The overall prevalence, awareness, treatment and control of hypertension according to selected population characteristics

	Hypertension	Awareness among hypertensive participants	Treatment among hypertensive participants	Treatment among hypertensive participants who were aware	Control among hypertensive participants	Control among hypertensive participants who were aware	Control among treated participants
	N=5409	N=2837	N=2022	N= 2022	N=861	N=861	N=621
	Prevalence (95% Confidence Interval)						
Overall	47.9 (47.0- 49.0)	53.2 (51.9-54.5)	38.2 (36.9 -39.5)	72.3 (70.6 – 73.9)	15.9 (14.9-16.9)	30.3 (28.7 – 32.0)	30.7 (28.7 -32.7)
Location							
Urban	44.9 (43.6 – 46.2)	54.1 (52.2 – 56.1)	42.0 (40.1 – 43.9)	78.4 (76.2 – 80.6)	18.9 (17.4 – 20.4)	35.6 (33.1 – 38.1)	36.5 (33.7 – 39.4)
Rural	51.2 (49.8 – 52.4)	52.3 (50.5 – 54.2)	34.7 (32.9 – 36.4)	66.4 (63.9 – 68.8)	13.1 (11.8 – 14.4)	25.2 (22.9 – 27.5)	24.2 (21.4 – 26.9)
* P-*value	< 0.001*	0.190	<0.001*	<0.001*	<0.001*	< 0.001*	<0.001*
Gender							
Male	50.9 (49.5 – 52.3)	49.5 (47.6 – 51.5)	35.9 (34.0 – 37.8)	73.0 (70.5 – 75.5)	14.2 (12.8 – 15.5)	29.0 (26.5 – 31.6)	30.4 (27.3 – 33.4)
Female	45.6 (44.3 – 46.8)	56.4 (54.6 -58.2)	40.2 (38.4 – 42.0)	71.7 (69.5 – 73.9)	17.4 (16.1 – 18.8)	31.3 (29.1 – 33.6)	31.0 (28.3 – 33.7)
* P-*value	<0.001*	<0.001*	0.001*	0.428	0.001 *	0.185	0.757
Age (years)							
30-39	19.1 (16.9 – 21.3)	35.3 (29.2 – 41.4)	19.6 (14.5 – 24.7)	55.4 (44.7 – 66.2)	13.1 (8.8 – 17.5)	37.3 (26.9 – 47.8)	37.0 (22.8 – 51.1)
40-49	35.7 (34.0 – 37.3)	48.0 (45.1 – 50.9)	29.5 (26.9 – 32.1)	61.8 (57.8 – 65.9)	17.1 (14.9 – 19.2)	36.0 (32.0 – 40.0)	33.9 (28.9 – 38.9)
50-59	51.6 (49.9 – 53.2)	53.2 (50.9 – 55.5)	39.1 (36.9 – 41.4)	74.1 (71.3 – 76.8)	16.2 (14.5 – 17.9)	30.7 (27.8 – 33.6)	31.9 (28.5 – 35.3)
≥ 60	68.2 (66.6 – 69.8)	58.1 (56.1 – 60.3)	44.4 (42.3 – 46.6)	76.8 (74.4 – 79.2)	15.4 (13.8 – 16.9)	26.9 (24.5 – 29.5)	28.3 (25.3 – 31.2)
* P-*value	< 0.001*	< 0.001*	< 0.001*	<0.001*	0.373	0.001*	0.137
Ethnic Group							
Malays	48.3 (47.2 – 49.3)	55.8 (54.2 -57.3)	40.4 (38.9 – 42.0)	73.0 (71.2 – 74.9)	16.1 (15.0 -17.3)	29.3 (27.4 – 31.3)	29.4 (27.2 -31.7)
Chinese	47.5 (44.7 – 50.3)	52.3 (48.2 – 56.4)	43.6 (39.5 – 47.7)	83.4 (79.2 – 87.7)	16.3 (13.3 – 19.3)	31.8 (26.4 – 37.1)	32.4 (26.5 – 38.2)
Indians	44.9 (39.6 – 50.4)	51.4 (43.2 – 59.5)	40.4 (32.4 – 48.4)	78.7 (69.3 – 88.0)	21.8 (15.1 – 28.5)	42.7 (31.4 – 53.9)	45.8 (32.9 – 58.6)
Indigenous	47.0 (44.5 – 49.5)	40.4 (36.8 – 44.0)	21.6 (18.6 – 24.6)	53.6 (47.9 – 59.3)	13.2 (10.8 – 15.7)	33.2 (27.8 – 38.6)	35.3 (27.7 – 42.8)
*P-*value	0.546	<0.001*	<0.001*	<0.001*	0.048*	0.050	0.025*
Education attainment							
No formal education	57.3 (54.9 – 59.8)	51.2 (48.0 – 54.5)	33.6 (30.4 – 36.7)	65.6 (61.3 -70.0)	14.3 (11.8 – 16.3)	27.5 (23.4 – 31.5)	29.0 (23.9 – 34.1)
Primary	58.7 (56.9 – 60.6)	58.2 (55.8 – 60.6)	42.7 (40.3 – 45.1)	73.7 (70.9 – 76.6)	14.7 (13.0 – 16.4)	25.3 (22.5 – 28.1)	25.9 (22.6 – 29.1)
Secondary	45.1 (43.5 – 46.6)	50.7 (48.3 – 53.0)	37.1 (34.8 – 39.4)	73.8 (70.9 – 76.7)	16.2 (14.5 – 17.9)	32.1 (29.1 – 35.2)	30.8 (27.3 – 34.4)
Tertiary	39.1 (36.9 – 41.2)	52.1 (48.6 – 55.7)	38.0 (34.5 – 41.4)	73.0 (68.6 -77.4)	21.2 (18.3 -24.1)	40.8 (36.0 – 45.6)	42.5 (36.8 – 48.1)
* P-*value	<0.001*	<0.001*	<0.001*	0.007*	<0.001*	< 0.001*	<0.001*
Smoking status							
Never smoke	49.5 (48.4 – 50.6)	53.5 (52.0 – 55.1)	39.2 (37.6 – 40.7)	73.7 (71.8 – 75.6)	16.0 (14.8 – 17.1)	29.9 (28.0 – 31.9)	30.1 (27.8 – 32.4)
Current smoker	42.3 (39.7 – 45.0)	48.0 (43.9 – 52.1)	30.1 (26.4 – 33.9)	63.1 (57.4 - 68.9)	14.0 (11.2 – 16.8)	29.2 (23.9 – 34.6)	28.9 (22.1 – 35.7)
Ex-smoker	58.3 (55.5 – 61.0)	55.3 (51.6 – 58.9)	39.2 (35.6 – 42.8)	71.1 (66.6 -75.6)	17.7 (14.9 – 20.5)	32.1 (27.4 – 36.7)	34.2 (28.6 – 39.8)
*P-*value	<0.001*	0.022*	<0.001*	<0.001*	0.199	0.656	0.356
Body Mass Index							
Normal	39.3 (37.4 – 41.2)	46.7 (43.5 – 49.8)	30.2 (27.3 – 33.1)	65.1 (60.6 -69.5)	15.0 (12.8 – 17.3)	32.7 (28.3 – 37.0)	32.2 (26.8 – 37.6)
Underweight	31.4 (26.8 – 36.0)	37.0 (28.3 – 45.7)	20.2 (12.9 – 27.4)	54.4 (39.7 -69.4)	11.4 (5.7 – 17.0)	31.8 (17.9 – 45.7)	29.2 (10.6 – 47.8)
Overweight	48.6 (47.1 – 50.1)	50.4 (48.3 – 52.7)	36.1 (34.0 – 38.2)	72.1 (69.3 – 74.9)	15.8 (14.2 – 17.4)	31.8 (28.9 – 34.7)	31.6 (28.2 – 35.0)
Obese	60.6 (59.0 – 62.2)	59.5 (57.4 – 61.6)	44.6 (42.6 – 46.8)	75.4 (73.0 – 77.7)	16.4 (14.8 – 17.9)	27.8 (25.4 – 30.2)	29.1 (26.2 – 31.9)
*P-*value	< 0.001*	< 0.001*	< 0.001*	< 0.001*	0.426	0.108	0.627

* significant at *p* < 0.05

In terms of locality, there was a significantly higher prevalence of hypertension in the rural than in urban areas (51.2 % vs. 44.9 %, *p* < 0.001). However, there was a significantly lower prevalence of treatment (34.7 % vs. 42.0 %, *p* < 0.001) and control (13.1 % vs. 18.9 %, *p* < 0.001) among hypertensive participants in the rural compared to urban areas, respectively. Among those who were aware, there was also a significantly lower prevalence of treatment (66.4 % vs. 78.4 %, *p* < 0.001) and control (25.2 % vs. 35.6 %, *p* < 0.001) in the rural compared to urban areas. Out of those who were treated, there was also a significantly lower prevalence of control (24.2 % vs. 36.5 %, *p* < 0.001) in the rural compared to urban areas.

Males had a significantly higher prevalence of hypertension compared to females, (50.9 % vs. 45.6 %, *p* < 0.001). However, a significantly lower prevalence of awareness (49.5 % vs. 56.4 %, *p* < 0.001), treatment (35.9 % vs. 40.2 %, *p* = 0.001) and control (14.2 % vs.17.4 %, *p* = 0.001) were observed in males compared to females among hypertensive participants. With regards to age, participants aged ≥ 60 years had the highest prevalence of hypertension (68.2 %), awareness (58.1 %) and treatment (44.4 %) compared to the other age groups.

There was no significant difference of prevalence of hypertension among the ethnic groups. Malays had the highest prevalence of awareness (55.8 %) while Chinese had the highest prevalence of treatment (43.6 %) compared to other ethnic groups. In contrast, the Indigenous group had the lowest prevalence of awareness (40.4 %) and treatment (21.6 %) compared to other ethnic groups. These trends were significant.

Participants with primary education attainment had the highest prevalence of hypertension (58.7 %), awareness (58.2 %) and treatment (42.7 %) compared to those in other education levels. Meanwhile, those with tertiary education level had the highest prevalence of control (21.2 %) compared to those in other education levels. These trends were significant. Ex- smokers had the highest prevalence of hypertension (58.3 %). In contrast, current smokers had the lowest prevalence of awareness (48.0 %) and treatment (30.1 %) compared to non-smokers or ex-smokers. Obese participants had the highest prevalence of hypertension (60.6 %), awareness (59.5 %) and treatment (44.6 %) compared to those in other BMI categories.

Socio-demographic factors associated with hypertension, awareness, treatment and control

Table 3 shows factors associated with hypertension, awareness, treatment and control among treated hypertensive participants. In a modified Poisson regression model that controlled for location, gender, age, ethnicity, education level, smoking status and BMI, the independent factors associated with hypertension were residing in the rural areas, female, age, Chinese, tertiary education, current smoker and all BMI categories. Meanwhile, the factors associated with awareness among the hypertensive participants were female, age, Indigenous groups and being obese. Factors associated with treatment among hypertensive participants were female, age, Indigenous groups and being overweight or obese. With regards to factors associated with control among treated participants, residing in the rural areas, female and Indian were identified as the independent factors.

**Table 3:** Factors associated with prevalence of hypertension, awareness, treatment and control among treated hypertensive participants

Risk Factors	Hypertension		Awareness among hypertensive participants		Treatment among hypertensive participants		Control among treated participants	
	CPR (CI)	APR† (CI)	CPR (CI)	APR† (CI)	CPR (CI)	APR† (CI)	CPR (CI)	APR† (CI)
Location								
Urban	1.00	1.00	1.00	1.00	1.00	1.00	1.00	1.00
Rural	1.14 (1.08–1.20)**	1.12 (1.04–1.20)*	0.97 (0.9–1.04)	1.03 (0.94–1.13)	0.83 (0.76–0.90)**	0.95 (0.85–1.06)	0.66 (0.56–0.78)**	0.61 (0.49–0.75)**
Gender								
Male	1.00	1.00	1.00	1.00	1.00	1.00	1.00	1.00
Female	0.90 (0.85–0.95)**	0.88 (0.83–0.94)**	1.14 (1.06–1.23)*	1.19 (1.08–1.31)**	1.12 (1.02–1.22)*	1.13 (1.01–1.26)*	1.02 (0.87–1.2)	1.25 (1.01–1.54)*
Age (years)								
30–39	1.00	1.00	1.00	1.00	1.00	1.00	1.00	1.00
40–49	1.86 (1.62–2.14)**	1.71 (1.48–1.98)**	1.36 (1.08–1.71)*	1.30 (1.03–1.65)*	1.51 (1.11–2.05)*	1.50 (1.09–2.07)*	0.92 (0.55–1.53)	0.92 (0.54–1.55)
50–59	2.70 (2.35–3.09)**	2.37 (2.06–2.74)**	1.51 (1.2–1.89)**	1.45 (1.15–1.83)*	2.00 (1.48–2.69)**	1.98 (1.45–2.70)**	0.86 (0.53–1.41)	0.85 (0.51–1.42)
≥60	3.56 (3.12–4.08)**	3.17 (2.74–3.67)**	1.65 (1.32–2.06)**	1.63 (1.29–2.06)**	2.27 (1.69–3.05)**	2.36 (1.73–3.24)**	0.77 (0.47–1.25)	0.77 (0.46–1.31)
Ethnicity								
Malay	1.00	1.00	1.00	1.00	1.00	1.00	1.00	1.00
Chinese	0.99 (0.9–1.08)	1.12 (1.01–1.24)*	0.94 (0.83–1.06)	1.01 (0.88–1.16)	1.08 (0.94–1.24)	1.13 (0.97–1.32)	1.10 (0.87–1.40)	0.90 (0.69–1.18)
Indian	0.93 (0.79–1.1)	1.04 (0.87–1.24)	0.92 (0.73–1.16)	0.95 (0.74–1.22)	1.00 (0.77–1.30)	1.00 (0.76–1.33)	1.56 (1.06–2.29)*	1.35 (0.89–2.04)
Indigenous groups	0.97 (0.9–1.05)	1.04 (0.95–1.14)	0.72 (0.64–0.82)**	0.80 (0.69–0.92)*	0.53 (0.45–0.63)**	0.66 (0.55–0.79)**	1.20 (0.91–1.59)	1.64 (1.19–2.25)*
Education attainment								
No formal education	1.00	1.00	1.00	1.00	1.00	1.00	1.00	1.00
Primary	1.03 (0.94–1.11)	1.00 (0.92–1.1)	1.14 (1.02–1.27)*	1.09 (0.96–1.22)	1.27 (1.11–1.46)*	1.15 (1.00–1.33)	0.89 (0.69–1.15)	1.03 (0.78–1.35)
Secondary	0.79 (0.73–0.85)**	0.94 (0.85–1.03)	0.99 (0.88–1.11)	0.99 (0.86–1.12)	1.11 (0.96–1.27)	1.05 (0.9–1.23)	1.06 (0.83–1.37)	1.03 (0.77–1.38)
Tertiary	0.68 (0.62–0.75)**	0.86 (0.76–0.96)*	1.02 (0.89–1.16)	1.05 (0.89–1.23)	1.13 (0.96–1.33)	1.07 (0.89–1.3)	1.46 (1.11–1.93)*	1.30 (0.94–1.81)
Smoking status								
Never smoke	1.00	1.00	1.00	1.00	1.00	1.00	1.00	1.00
Current smoker	0.86 (0.78–0.93)**	0.81 (0.73–0.89)**	0.90 (0.79–1.02)	0.99 (0.86–1.14)	0.77 (0.66–0.90)*	0.84 (0.7–1.00)	0.96 (0.72–1.28)	1.11 (0.79–1.55)
Ex-smoker	1.18 (1.09–1.27)**	1.00 (0.91–1.09)	1.03 (0.93–1.15)	1.12 (0.98–1.26)	1.00 (0.88–1.14)	1.04 (0.9–1.21)	1.13 (0.91–1.41)	1.28 (0.99–1.67)
Body Mass Index								
Normal	1.00	1.00	1.00	1.00	1.00	1.00	1.00	1.00
Underweight	0.80 (0.66–0.96)*	0.72 (0.59–0.89)*	0.79 (0.58–1.08)	0.77 (0.56–1.08)	0.67 (0.44–1.01)	0.69 (0.45–1.07)	0.91 (0.42–1.95)	1.31 (0.6–2.87)
Overweight	1.24 (1.15–1.34)**	1.31 (1.2–1.42)**	1.08 (0.97–1.21)	1.10 (0.98–1.24)	1.20 (1.04–1.37)*	1.19 (1.03–1.38)*	0.98 (0.77–1.25)	0.95 (0.74–1.23)
Obese	1.54 (1.43–1.66)**	1.71 (1.58–1.86)**	1.28 (1.15–1.42)**	1.30 (1.16–1.46)**	1.48 (1.3–1.69)**	1.51 (1.31–1.74)**	0.90 (0.72–1.14)	0.88 (0.68–1.13)

†Modified Poisson regression model

CPR: Crude prevalence ratio, APR: Adjusted prevalence ratio, CI: 95 % confidence interval

*significant at *p* < 0.05; **significant at *p* < 0.001

## Discussion

The REDISCOVER Study provided evidence that nearly one in two (overall prevalence = 47.9 %; age-adjusted prevalence = 42.0 %) Malaysians over 30 years of age were hypertensive and its treatment and control were still inadequate. Using the same age cut off ≥ 30 years old, this study showed a rising overall prevalence of hypertension compared to the NHMS 2011 (43.5 %) [4], NHMS III 2006 (42.6 %) [11] and NHMS II 1996 (32.9 %) [5]. In addition, when comparing Malaysia to the other South East Asian countries, Malaysia had the highest prevalence of hypertension among adults ≥ 18 years (32.7 %) according to NHMS 2011 [4] compared to Singapore (26.6 %), Indonesia (23.%) and Thailand (20.5 %) [12]. A similar rising trend is also observed in the prevalence of other associated cardiovascular risk factors among the Malaysian population e.g. obesity and dyslipidaemia [6]. Urbanization phenomenon, sedentary lifestyle, high consumption of salt and fatty food may have contributed to the rising prevalence of hypertension and the associated cardiovascular risk factors in Malaysia [13].

With regards to the awareness rate in Malaysian adults ≥ 30 years of age, REDISCOVER showed a higher awareness (53.2 %) compared to NHMS 2011 (42.5 %) [4], NHMS III (35.8 %) [11] and NHMS II (33.0 %) [5]. Nevertheless, a much higher rate of awareness was observed in a developed country like the USA (82.9 % in men and 80.3 % in women ≥ 30 years) [14] and also amongst the rural community in Thailand (64.9 %) [15]. Despite an improved awareness, the treatment rate observed in this study was lower when compared to Thailand (38.2 vs. 42.6 %) [15] and has not changed much compared to an earlier local study (32.4 %) [3]. However, the treatment rate was comparable to countries like China (34.1 %) [16] and Turkey (37.7 %) [17]. Among hypertensive participants in this study who were aware of their hypertension status, the treatment rate (72.3 %) was comparable to the finding in NHMS 2011 (78.4 %) [4].

An improved overall control rate was observed in this study (15.9 %) when compared to NHMS III (8.2 %) [11] and NHMS II (6 %) [5]. Similarly, this study showed an improvement in the control rate amongst those on treatment (30.7 %) compared to NHMS III and II which showed a stagnant rate of 26.0 % [5, 11]. The improvements in the awareness, treatment and control rates over the last decades may have been attributed to the various public health and primary care measures mooted by the Health Ministry. These include continuous enhancement of the public primary care services, as well as improvements in the availability of antihypertensive agents in health clinics where majority of the hypertensive individuals are managed. Nevertheless, efforts to heighten public awareness, treatment and control should be continued and further enhanced.

Globally, the prevalence of hypertension in the urban adult populations were between 15–35 % but was found to be lower in the rural Asian populations [18]. Interestingly, in comparing the urban and rural populace in this study, there was a higher prevalence of hypertension in the rural compared to the urban population (51.2 % vs. 44.9 %). Rural population was found to be more likely to have hypertension (APR: 1.12, CI: 1.04–1.20). This is in contrast to studies in Africa [19, 20] and India [21] where higher prevalence of hypertension were observed in their urban populations. The higher prevalence of hypertension in the rural population in this study could be explained by lifestyle factors such as physical inactivity, excess dietary intake of sodium and fat as well as obesity which have spread from urban to rural areas at an alarming rate [13]. A strong correlation between urbanization and prevalence of hypertension was also evidenced in India, where a sharp increase in per capita net domestic product, growth production and human development indexed correlated positively with the hypertension increase in rural areas [22]. A significantly lower control rates in the rural areas were observed in this study which were similar to other studies in China and Thailand [23, 24]. Rural population was less likely to achieve control (APR 0.61; CI: 0.49–0.75) compared to their urban counterparts. Poorer BP control in the rural participants may be due to lack of knowledge on hypertension and disparity in healthcare services available in the rural compared to urban areas. The geographical condition of East Malaysia that is still preserved with the tropical rain forest and rivers as the main transportation method remains a challenge in establishing good access to healthcare. The other possibility is the use of complementary and alternative medicines as part of self-management and this was found to be prevalent in rural areas [25]. This study highlights the need to put more efforts to reduce the prevalence and increase the control of hypertension in the rural areas of Malaysia.

In evaluating gender differences, a higher age adjusted prevalence of hypertension was observed in males than in females. However, females had a significantly higher prevalence of awareness, treatment and control which were consistent with other studies [15, 26]. In the regression analyses, females were less likely to have hypertension (APR 0.88; CI: 0.83–0.94) but they were more likely to be aware (APR 1.19; CI: 1.08–1.31), to be treated (APR 1.13; CI: 1.01–1.26) and to achieve BP control once treated (APR 1.25; CI: 1.01–1.54) when compared to males. This data suggests the importance of raising hypertension awareness as well as controlling hypertension in men. A recent review highlighted the importance of promoting and improving men’s health in the country due to a higher prevalence of cardiovascular risk factors and an increasing cancer related death among men [27]. Barriers to health seeking behaviour among men include lack of time to adopt healthy lifestyle, men’s enjoyment to be among fellow smokers and men’s perception of invulnerability to illness [28]. More studies are needed to identify effective strategies to improve men’s health seeking behavior and efforts to improve men’s health should be in place.

Published evidences have shown that older age was associated with a higher prevalence, awareness and treatment of hypertension [29–31] which were consistent with findings from REDISCOVER. Participants aged ≥ 60 years old were found to be three times more likely to have hypertension (APR 3.17; CI 2.74–3.67), almost twice more likely to be aware (APR 1.63; CI: 1.29–2.06) and twice more likely to be on treatment (APR 2.36; CI 1.73–3.24) compared to participants aged 30–39 years in the study. It was postulated that older people who are at higher cardiovascular risk have better awareness and hence receive treatment more than younger individuals. It is concerning to note that younger individuals were less aware of their hypertension status and were less likely to be on antihypertensive treatment. Hence, it is important to educate younger individuals to have regular health screening and to seek treatment early in order to minimize cardiovascular complications.

There was no significant difference in prevalence of hypertension among the ethnic groups in this study. Earlier studies conducted locally noted that all ethnic groups were found to have uniformly high prevalence of hypertension [2, 3]. REDISCOVER also found that the Indigenous groups who resided in the remote areas were less likely to be aware (APR 0.8; CI: 0.69–0.92) and to be on treatment (APR 0.66; CI: 0.55–0.79) for hypertension compared to the Malays.

This study shows that participants with primary education level had the highest prevalence of hypertension, awareness and treatment, while those with tertiary education level had the highest prevalence of control. Those with tertiary education level were also found to be less likely to have hypertension (APR 0.86; CI: 0.76–0.96). This trend was also found in another study [32]. Those with lower education attainment may be leading a less healthy lifestyle to explain the higher prevalence of hypertension, and may have a lack of knowledge on the importance of good hypertension control. This finding underlines the needs to deliver better patient education targeting those with lower education level.

Ex-smokers had a significantly higher prevalence of hypertension than non-smokers or current-smokers. This finding was supported by other study which found that the prevalence of hypertension was higher in former smokers than non-smokers and the risk was associated with higher body mass and higher prevalence of obesity in ex-smokers [33]. However, further regression analyses found that current smokers were found to be less likely (APR 0.81; CI: 0.73–0.89) to have hypertension compared to non-smokers. Further studies are needed to understand the effects of smoking status and hypertension. Nonetheless, smoking was identified as a risk factor for development of hypertension [34].

Overweight and obese participants in this study were found to have the highest prevalence of hypertension compared to those in the other BMI categories. Participants who were overweight (APR 1.31; CI: 1.20–1.42) and obese (APR 1.71; CI: 1.58–1.86) were found to be more likely to have hypertension, which support the postulation that obesity are risk factors for hypertension [16, 32, 35]. It was reported that Asian populations are more inclined to obesity with increased insulin resistance compared to their Western counterparts [36]. Hence, with increasing globalization together with hazardous behaviour which are highly prevalent in Malaysia e.g. smoking [37], high fat intake and low physical activity [13, 38]; concerted efforts need to be taken to reduce these modifiable risk factors in our population.

REDISCOVER gave insight on the current magnitude of hypertension burden in the Malaysian population. Higher hypertension prevalence would translate into higher number of individuals with future cardiovascular diseases and events; leading to increased utilization of healthcare services, escalating health care costs, increased premature deaths, reduced productivity and increased economic burden [1, 39]. Public health measures, as well as individual interventions in primary care are crucial to reduce this rising epidemic. Public health measures should include multi-sectorial collaborations involving relevant stakeholders in the community. At the primary care level, individual patients aged ≥ 30 years old should be routinely screened for hypertension and its associated cardiovascular risk factors at regular intervals and at opportunistic times. Special attention should be given to groups most affected and those who were least aware, treated and controlled. These include the rural population, male participants, Indigenous group, younger, overweight and obese individuals. Programs may incorporate intervention to improve the knowledge, attitude and behaviours of patients and health care professionals in order to diagnose hypertension early and improve adherence to treatment to achieve better control and prevent complications.

This study has several limitations. First, comparisons in the prevalence of hypertension, awareness, treatment and control rates with other countries may be hindered by the different methodologies and criteria used between studies. Second, information bias may have been present with respect to both recall diagnosis of hypertension and treatment. However, proper training of all manpower involved in conducting the survey and its procedures minimized potential bias. Third, two measurements of BP on a single visit were used to diagnose hypertension in this study as opposed to the recommendation by the Malaysian CPG of Management of Hypertension, 3^rd^ edition, 2008, which recommended BP measurements on at least 2 separate visits for diagnosis [40]. However, similar method was used to diagnose hypertension in the other national surveys and several other studies due to the difficulties to measure BP on 2 separate visits in large population surveys [3, 41]. Fourth, Malays were over represented, while Chinese and Indian populations were under represented in this study as Malaysia’s ethnic population comprises 53.3 % Malays, 26.0 % Chinese, 7.7 % Indians and the remaining 13 % are of other ethnic groups [7]. Our study population also had more females and older individuals than the Malaysian population. This may be due to the fact that males and younger participants were working when screenings were conducted, or females were more health conscious and therefore, were more likely to attend health screening programmes. Hence, interpretation of the result should be done with caution. Nevertheless, the large sample size of this study population should counter balance any possible bias in the sampling.

## Conclusion

In conclusion, REDISCOVER highlights an alarming situation as almost half of Malaysian adults aged ≥ 30 years have hypertension and of those, only half were aware of their hypertension status, less than 40 % were on treatment and only a third of those were controlled. Comprehensive intervention strategies that target the general population, as well as focus on improving awareness, treatment and control among rural population, men, younger individuals, Indigenous groups, overweight and obese individuals must be put in place. Findings from this study emphasize the urgency to stem the rising tide of hypertension prevalence and the almost inevitable emerging epidemic of cardiovascular diseases in Malaysia. Public health measures, as well as individual interventions in primary care are crucial to reduce their risk of developing complications.

## Availability of data and materials

Data are kept at the Centre for Translational Research and Epidemiology (CenTRE), Faculty of Medicine, Universiti Teknologi MARA. Data will be shared upon request and it is subjected to the data protection regulations.

## Abbreviations

APR: adjusted prevalence ratio
BMI: body mass index
BP: blood pressure
CI: confidence interval
CPR: crude prevalence ratio
NHMS: National Health Morbidity Survey
SD: standard deviation
